# Supplementary material for: ABPP-HT*—Deep Meets Fast for Activity-Based Profiling of Deubiquitylating Enzymes Using Advanced DIA Mass Spectrometry Methods
Source: Int J Mol Sci. 2022 Mar 17;23(6):3263. doi: 10.3390/ijms23063263 (PMC8955990; doi:10.3390/ijms23063263)
Supplement: Supplementary file 1 [file ijms-23-03263-s001.zip › ijms-1627256-supplementary.pdf]

Figure S1

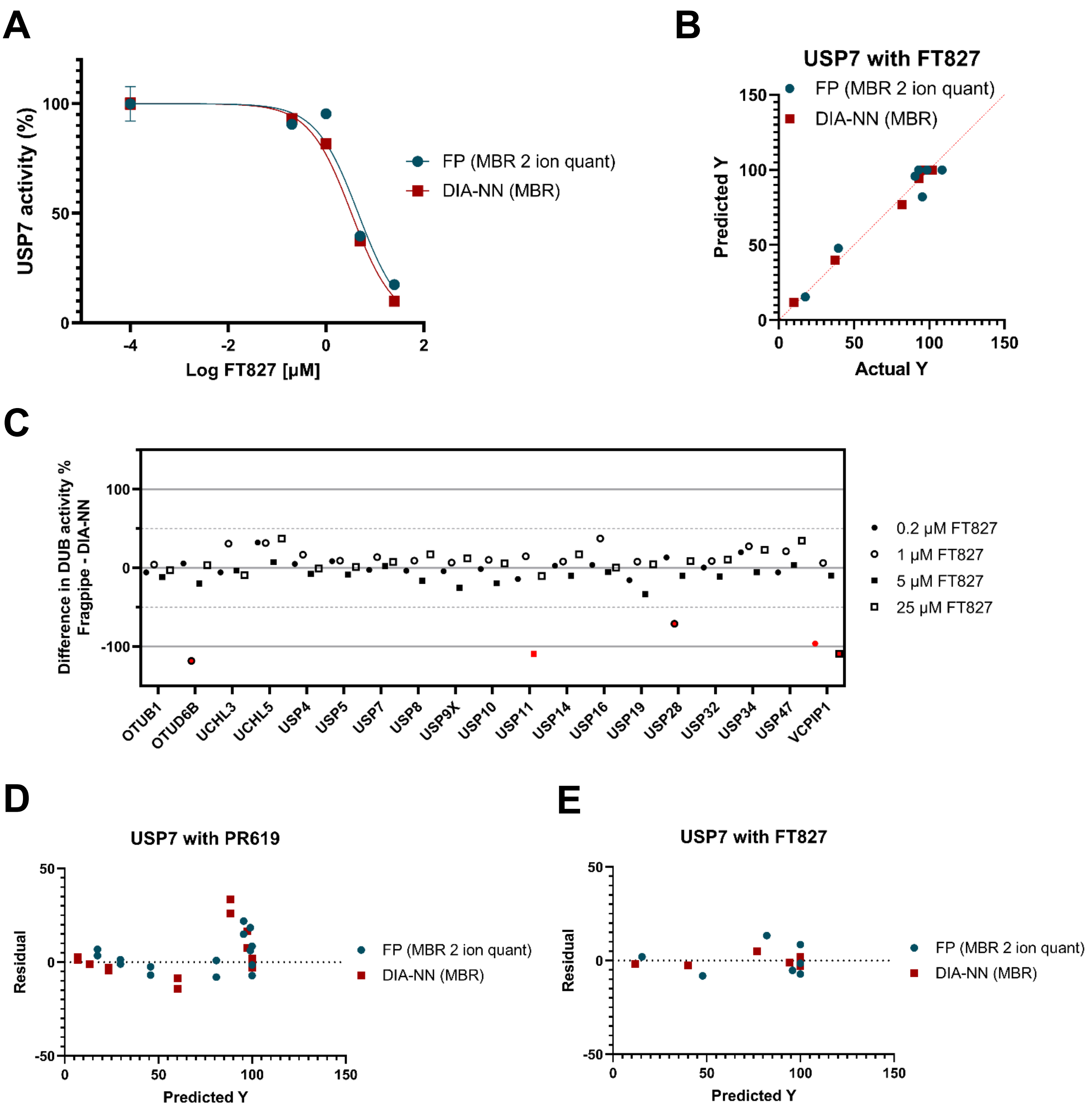

Supplementary Figure S1. **A)** USP7 IC_{50} curve showing reduction of USP7 intensity with increasing FT827 concentrations, collected as either DIA data analysed with DIA-NN MBR* or DDA data Fragpipe data 2 ion quant MBR* fit to Y=100/(1+10^((X-LogIC_{50}))). *MBR as part of a larger dataset with other inhibitors. **B)** Predicted vs actual Y values of the IC_{50} curve shown in A. **C.** Difference plot of common DUBs identified by both DIA-NN MBR and Fragpipe 2 ion quant MBR with DIA-NN % activity subtracted from Fragpipe % activity at various FT827 concentrations. Values in red are attributed to missing

values and not as a result of inhibition differences due to their reduction not occurring in a concentration dependent manner with increasing inhibitor concentration. **D)** Residual vs predicted Y for USP7 with PR619 IC_{50} fit. **E)** Residual vs predicted Y for USP7 with FT827 IC_{50} fit.

Supplementary Table 1

Start m/z	End m/z	Start 1/k0	End 1/k0
400	425	0.6	0.834
425	450	0.6	0.858
450	475	0.6	0.883
475	500	0.6	0.908
500	525	0.6	0.932
525	550	0.6	0.957
550	575	0.6	0.982
575	600	0.6	1.005
600	625	0.835	1.031
625	650	0.858	1.055
650	675	0.884	1.079
675	700	0.909	1.105
700	725	0.934	1.129
725	750	0.958	1.153
750	775	0.983	1.179
775	800	1.006	1.202
800	825	1.032	1.638
825	850	1.056	1.638
850	875	1.08	1.638
875	900	1.106	1.638
900	925	1.13	1.638
925	950	1.154	1.638
950	975	1.18	1.638
975	1000	1.204	1.638

Supplementary Table 1

DIA-PASEF windows for data in Figures 1, 2 ,3C - E + 4.

Supplementary Table 2

Start m/z	End m/z	Start 1/k0	End 1/k0
475	500	0.85	0.89
500	525	0.85	0.91
525	550	0.85	0.93
550	575	0.85	0.95
575	600	0.85	0.97
600	625	0.85	0.99
625	650	0.85	1.01
650	675	0.87	1.03
675	700	0.89	1.04
700	725	0.91	1.06
725	750	0.93	1.08
750	775	0.95	1.1
775	800	0.97	1.12
800	825	0.99	1.14
825	850	1.01	1.16
850	875	1.03	1.18
875	900	1.05	1.2
900	925	1.07	1.22
925	950	1.08	1.24
950	975	1.1	1.26
975	1000	1.12	1.27

Supplementary Table 2

DIA-PASEF windows for data in Figures 3A + B.
